# Tsantan Sumtang Restored Right Ventricular Function in Chronic Hypoxia-Induced Pulmonary Hypertension Rats

**DOI:** 10.3389/fphar.2020.607384

**Published:** 2021-01-18

**Authors:** Zhanting Yang, Haixia Sun, Shanshan Su, Xingmei Nan, Ke Li, Xueqin Jin, Guoen Jin, Zhanqiang Li, Dianxiang Lu

**Affiliations:** ^1^ Research Center for High Altitude Medicine, Key Laboratory of High Altitude Medicine (Ministry of Education), Key Laboratory of Application and Foundation for High Altitude Medicine Research in Qinghai Province (Qinghai-Utah Joint Research Key Lab for High Altitude Medicine), Qinghai University, Xining, China; ^2^ Department of Cardiac Ultrasound, Qinghai Provincial People’s Hospital, Xining, China; ^3^ Technical Center of Xining Customs, Key Laboratory of Food Safety Research in Qinghai Province, Xining, China; ^4^ Laboratory Animal Center, Ningxia Medical University, Ningxia, China

**Keywords:** Tsantan Sumtang, right ventricular function, right ventricular-pulmonary arterial coupling, rho kinase (ROCK) signaling pathway, chronic hypoxia-induced pulmonary hypertension

## Abstract

**Background:** Tsantan Sumtang originated from *Four Tantras,* which consisted of *Choerospondias axillaris* (Roxb.) B. L. Burtt and A. W. Hill, *Santalum album* L., and *Myristica fragrans* Houtt. The three herbs are in ratio 1:1:1. This medication is widely used for cardiovascular diseases.

**Aims:** The purpose of this study was to explore the effect of Tsantan Sumtang on right ventricular (RV) function in hypoxia-induced pulmonary hypertension (HPH) rats and investigate the underlying mechanism.

**Methods:** Sixty male Sprague-Dawley (SD) rats were divided into control, hypoxia, and hypoxia + Tsantan Sumtang (1.0, 1.25, and 1.5 g•kg^−1^•d^−1^) groups. Chronic hypoxia was induced by putting the rats inside a hypobaric chamber for four weeks and adjusting the inner pressure and oxygen content to match an altitude of 4500 m. Echocardiography was used to assess RV function and right ventricular-pulmonary arterial (RV-PA) coupling. The physiological parameters of the animals were also evaluated. Morphological characteristics of RV were assessed by hematoxylin and eosin (H&E) staining and TEM. Masson’s trichrome staining, immunohistochemical staining, western blotting, and TUNEL assay were used to assess fibrosis and apoptosis levels. The antioxidant and anti-apoptosis properties of Tsantan Sumtang were also evaluated. The effect of Tsantan Sumtang on ROCK signaling pathway was evaluated using real-time quantitative PCR and western blotting.

**Results:** We established an HPH rat model as indicated by the significant increases in the physiological parameters of the rats. Tsantan Sumtang showed a significant cardiac-protective function and an improved effect on RV-PA coupling. Moreover, Tsantan Sumtang treatment inhibited fibrosis and alleviated apoptosis and oxidative stress in RV. In terms of mechanism, Tsantan Sumtang reduced the expression of ROCK (ROCK1, ROCK2) in RV, inhibited cardiac remodeling-related transcription factors (NFATc3, P-STAT3), and regulated apoptosis-related proteins.

**Conclusion:** Tsantan Sumtang was able to restore RV function, improve RV-PA coupling, recover hemodynamic and hematological indexes, and protect RV against structural maladaptive remodeling in the HPH rats. These findings demonstrated that Tsantan Sumtang protects the function of RV in HPH rats. The antioxidant and anti-apoptosis properties of Tsantan Sumtang may be responsible for inhibiting the ROCK signaling pathway.

## Introduction

Pulmonary arterial hypertension (PAH) is a clinical syndrome with a poor prognosis, characterized by elevated pulmonary artery pressure (de Jesus Perez, 2016). PAH causes pressure overload of the right ventricle and ultimately results in RV maladaptation or RV failure, which was the important factor of symptomatology and outcome in patients with PAH (Vonk Noordegraaf et al., 2017). Hypoxia is one of the reasons behind the onset and development of pulmonary hypertension (Naeije and Dedobbeleer, 2013). Exposure to chronic hypoxia causes pulmonary hypertension that is characterized by a progressive rise in pulmonary arterial pressure and pulmonary artery structural remodeling, which leads to RV hypertrophy. Prolonged hypoxia also affects cardiac myocytes, resulting in myocardial dysfunction (Naeije and Dedobbeleer, 2013). However, currently available vasodilator therapies are unable to sustainably reverse the pulmonary vascular changes or reduce the pulmonary artery pressure in the majority of PAH patients (Suen et al., 2019). A treatment strategy that maintains or improves RV structure and function in the face of elevated RV afterload might improve the survival of PAH patients (Tello et al., 2019a).

The pulsatile efficiency of RV relies on suitable hemodynamic coupling with compliant pulmonary circulation. The pulmonary vasculature resistance and compliance contribute to the RV afterload. Generally, the systolic function of RV dynamically adapts to the afterload, called RV-pulmonary arterial coupling (RV-PA coupling) (Aggarwal et al., 2018). In PAH patients, the right heart is adjusted to the increasing vascular load by enhancing contractility (“coupling”) to maintain flow (Grignola et al., 2007). Ventricular-arterial coupling implies that the stroke volume changes little while the ventricular efficiency is maintained. Optimal coupling between the right ventricle and pulmonary arterial load means that the maximum transfer of power from the ejecting chamber to the pulmonary artery is allowed (Grignola et al., 2007). PAH patients develop RV hypertrophy; RV contractility and RV-PA coupling are initially maintained (or increased) but then progressively decrease (He et al., 2016). RV structure and RV-PA coupling are necessary for RV function (Blumberg et al., 2013).

Tsantan Sumtang (Sanwei Tanxiang San), one of the traditional Tibetan medicines, consists of *Choerospondias axillaris* (Roxb.) B. L. Burtt and A. W. Hill (Roudoukou, Myristicae Semen), *Santalum album* L. (Tanxiang, Santali Albi Lignum), and *Myristica fragrans* Houtt (Guangzao, Fructus Choerospondiatis). *Four Tantras* mention this medicine as a traditional Tibetan medicine for “heart fever” (Luo et al., 2015; Yang et al., 2017). This medicine contains the fruit of *Choerospondias axillaris* (Roxb.) B. L. Burtt and A. W. Hill and *Myristica fragrans* Houtt, and the wooden heart of *Santalum album* L. The three herbs are in ratio 1:1:1. We previously showed that the aqueous extract of Tsantan Sumtang alleviated HPH in rats by inhibiting pulmonary vascular cell proliferation and suppressing cyclin D1 and CDK4 expression (He et al., 2018). Tsantan Sumtang also reduces hypoxia-induced RV remodeling and fibrosis by equilibrating ACE-AngII-AT1R and ACE2-Ang1-7-Mas axis of RV tissues of HPH rats (Dang et al., 2020). However, the effect of Tsantan Sumtang on the RV function of HPH rats is still unclear. Therefore, to elucidate the mechanism underlying the Tsantan Sumtang-mediated HPH alleviation in rats, we explored the effect of this medicine on the RV function in HPH rats.

## Materials and Methods

### Materials

Tsantan Sumtang (catalog # Z20020094) was purchased from Nei Monggol Kaimeng Pharmaceutical Co., Ltd. (Nei Monggol, Hohhot, China). The essential oil content was calculated using steam distillation. Rat N-terminal prohormone brain natriuretic peptide (NT-proBNP) ELISA kits were purchased from Elabscience Biotechnology Co., Ltd. (Hubei, Wuhan, China). Anti-collagen 1 (ab34710), anti-STAT3 (ab76315), anti-Bcl-2 (ab196495), anti-Bax (ab32503), anti-ROCK1 (ab45171), anti-ROCK2 (ab71598), and anti-beta actin (ab8226) were purchased from Abcam (Cambridge, Massachusetts, USA). Anti-cleaved caspase-3 (Asp175) was purchased from Cell Signaling Technology (Boston, USA). Anti-NFATc3 (18222-1-AP) was purchased from Proteintech (Chicago, USA). Proteinase K (Lot No. 1245680100) was purchased from Merck Millipore (Boston, USA). TUNEL kit (*in situ* cell death detection kit-POD method, Lot No. 10279600) was procured from Roche (Basel, Switzerland). Li Chunhong and phosphotungstic acid (Lot No. 20190115) was purchased from Chengdu Kelong Chemical Reagent Factory (Sichuan, Chengdu, China). Toluidine blue (Lot No. 190118) was purchased from Shanghai Ruji Biotechnology Development Company (China).

### Drug Identification

The total essential oil from 10 g of Tsantan Sumtang powder was extracted by the reflux extraction method for three times, 5 h per extraction. The essential oil content in 10 g Tsantan Sumtang was found to be 0.05 ml g^−1^ as evaluated by steam distillation (1 g of Tsantan Sumtang should contain more than 0.05 ml of essential oil as described in the Chinese Pharmacopoeia). The concentration of 3,4-dihydroxybenzoic acid was found to be 1.2 mg g^−1^ as detected with High-Performance Liquid Chromatography (HPLC) (column: C18, mobile phase: methanol:water (2.5% acetic acid) = 10:90, detection wavelength: 260 nm). The observations were accorded as the requirements of the Drug Standard (WS3-523(Z-100)-2005(Z)). Dehydrodiisoeugenol was also detected with HPLC (column: C18, mobile phase: methanol:water = 75:25, detection wavelength: 274 nm). The concentration of Dehydrodiisoeugenol was found to be 1.63 mg g^−1^. The constituents of essential oil were identified using gas chromatography-mass spectrometer analysis (GC-MS, Thermo Scientific, San Jose, CA, United States). Briefly, the method of GC-MS was as follows: programmed heating initial temperature 40°C, heating to 200° at 3°C min^−1^, then heated to 280°C at a rate of 8°C min^−1^, maintained for 5 min, injection volume 1.0 μL, no shunt injection, carrier gas (flow rate): helium (1.2 ml min^−1^), inlet temperature 250°C. The temperature of the ion transfer tube was 250°C, and the temperature of the ion source was 280°C. EI ion source was used as a mass spectrometry detector (70 eV, scan range: 50–600 m/z).

### Animals

This experimental protocol was approved by the Institutional Animal Care and Use Committee of Qinghai University in compliance with the animal management rules of the Chinese Ministry of Health. Rats were purchased from the Experimental Animal Center of Xi'an Jiaotong University, China (permit number SCXK (Shan) 2017-003). Sixty male Sprague-Dawley (SD) rats (*n* = 60, weight = 180 ± 20 g, 8 weeks old) were randomly divided into the following groups with twelve rats each: control, hypoxia, hypoxia + Tsantan Sumtang (1.0 g•kg^−1^•d^−1^), hypoxia + Tsantan Sumtang (1.25 g•kg^−1^•d^−1^), and hypoxia + Tsantan Sumtang (1.5 g•kg^−1^•d^−1^). For hypoxia group, rats were placed for four weeks in a hypobaric chamber (DYC-300, Guizhou Feng Lei Oxygen Chamber Co., Ltd., Guizhou, China) in which the pressure and oxygen content corresponded to that of 4500 m altitude. In all hypoxia + Tsantan Sumtang groups, rats were kept in the hypobaric chamber for four weeks and intragastrically administered with 1.0, 1.25, or 1.5 g•kg^−1^•d^−1^ of Tsantan Sumtang powder. Oxygen level, pressure, CO_2_ concentration, temperature, and relative humidity in the hypobaric chamber were 19.7%, 52.9 KPa, 1298 ppm, 18.0°C, and 46.9%, respectively.

### Echocardiography

Echocardiography was conducted using a Vevo 2100 imaging system (Visual Sonics, Toronto, Canada). Briefly, the chest hairs of the rats were removed, and the rats were anesthetized with 2% isoflurane/oxygen mixture with AS-01 step gas anesthesia machine (Northern Vaporiser Ltd., Britain) at room temperature. The RV inner diameter during diastole (RVID-Dia) was measured in the two-dimensional mode. Tricuspid annular plane systolic excursion (TAPSE) was measured from the apical 4-chamber view using two-dimensional and M-modes. Pulmonary artery acceleration time (PA-AT) was determined from the pulse wave Doppler interrogation at the pulmonary valve annulus in the parasternal short-axis view. RV end-systolic area (ESA) and RV end-diastolic area (EDA) were measured in the RV-focused apical 4-chamber. Fractional area change (FAC, %) was calculated as (EDA-ESA)/ESA×100%. Hemodynamic parameters were calculated after the echocardiographical test.

### Hemodynamic and Hematological Index Measurement

Rats were anesthetized with urethane (1.0 g•kg^−1^) through intraperitoneal injection, and their body weights were recorded. The mean pulmonary arterial pressure (mPAP) and systolic pulmonary arterial pressure (PASP) were determined by the right cardiac catheterization technique. A catheter was inserted through the right jugular vein, and it passed through the right ventricle to reach the pulmonary artery. The inserted catheter was positioned correctly using the waveform shown on the biological function experimental system (BL-420, Tai Meng Technology Co., Ltd., Chengdu, China). Right ventricle (RV), left ventricle (LV), and interventricular septum (S) of each rat were separated on the basis of the ventricular septal edge. The weight of them was measured to determine the right ventricle index (RV/LV + S, RVHI).

After hemodynamic analysis, 0.5 ml of the blood was collected from the abdominal aorta of each rat and transferred into ice-cold-heparinized sample vials for estimating various parameters using a blood cell counter (BC-5000 Vet, Mindray Company, Shenzhen, Nanshan, China). These parameters included red blood cell (RBC) count, hemoglobin (HGB) concentration, hematocrit (Hct), platelet (PLT) count, and white blood cell (WBC) count. Furthermore, plasma levels of NT-proBNP were detected using rat NT-proBNP ELISA kit.

### Morphometric Evaluation of RV Tissues

The RV tissues were fixed in 4% paraformaldehyde for 48 h, followed by paraffin embedding and sectioning (5 μm, RM2135 type paraffin microtome; Leica, Solms, Germany). The morphological changes were studied using hematoxylin and eosin (H&E) staining. RV tissues were stained with Masson's trichrome stain to assess the degree of fibrosis (collagen fibers stain blue). Images were acquired using a BA400 digital microscope (Motic China Group Co., Ltd., Xiamen, China) with the digital interface. TUNEL assay was used to detect apoptosis in the RV cells. Briefly, after the routine dewaxing procedure, we treated the sections with proteinase K solution at 37°C for 25 min. TUNEL reaction mixture (50 μL) was then added to the specimens after washing them thrice with PBS. The slides were covered and reacted in a dark wet box at 37°C for 1 h. Tissues were washed with PBS thrice and treated with 50 μL of Solution 3 (converter POD) at 37°C for 30 min in a dark wet box. After being washed again with PBS thrice, the tissues were treated with 50–100 μL DAB at 25°C for 10 min. The samples were slightly redyed by hematoxylin, after rinsing again with PBS thrice followed by washing the slides with tap water for seconds. The slides were sealed with neutral gum, after dehydrated by alcohol and transparented by xylene. An expert pathologist examined the ultrastructural changes in the RV tissues using a transmission electron microscope (H-600IV, Hitachi Limited, Tokyo, Japan).

### Immunohistochemical Staining for Collagen 1

Paraffin sections were prepared for immunohistochemical analysis using SP-HRP kits (SP-9000, ZSGB Biotechnology Co. Ltd., Beijing, China) according to the manufacturer’s instructions. The microwave heat-mediated method was used for the retrieval of antigenic sites. The sections were incubated with monoclonal mouse anti-collagen 1 (1:150, GTX11335, GeneTex, Texas, United States) antibody in Triton PBS (0.01 mmol/L) for 14 h at 4°C. The sections were then washed with PBS (three times for 3 min each) and incubated with biotinylated goat anti-mouse antibody for 15 min at 37°C. This was followed by washing the sections with PBS for 20 min and incubating the sections with horseradish peroxidase-conjugated streptavidin for 15 min at 37°C. The sections were visualized after staining them with diaminobenzidine for 10 min at room temperature and treating them with hematoxylin for 18 s.

### Antioxidant Activity Analysis

Malondialdehyde (MDA) content and superoxide dismutase (SOD) activity were estimated using thiobarbituric acid and xanthine oxidase methods, respectively. Glutathione (GSH) content and glutathione peroxidase (GSH-Px) activity were detected using dithiodinitrobenzoic acid and benzoic acid methods, respectively, according to the manufacturer's instructions (Nanjing Jiancheng, Jiangsu, Nanjing, China).

### Quantitative Real-Time PCR

Total RNA was extracted from frozen RV tissues of rats with TRIzol reagent (15596026, Invitrogen). cDNA was synthesized from 2000 ng RNA in a 20 μL reaction system using the Tiangen first-strand cDNA synthesis kit according to the manufacturer’s instructions (Tiangen Biotech Co., Ltd., Beijing, China). Quantitative real-time PCR (qRT-PCR) was performed inside the ABI7500 real-time PCR system (Bio-rad, California, USA) using Tiangen SYBR green supermix according to the manufacturer's instructions (Tiangen Biotech Co., Ltd., Beijing, China). β-actin was used as a loading control. The sequence information of all the primers used in this study is given in Table 1. The relative gene expression was calculated with the 2^−ΔΔCt^ method and normalized to β-actin.

**TABLE 1:** Oligonucleotide primers used for quantitative real-time PCR.

Gene	Primers	Nucleotide sequences 5’-3’	Length (bp)	Temp. (°C)
Bcl-2	Forward	GGG​CTA​CGA​GTG​GGA​TAC​TGG​AG	94	63.77
	Reverse	TCG​GTT​GCT​CTC​AGG​CTG​GAA​G		61.85
Bax	Forward	TCC​TCA​CTG​CCT​CAC​TCA​CCA​TC	121	61.99
	Reverse	CCT​TTC​CCC​GTT​CCC​CAT​TCA​TC		61.99
Cleaved Caspase-3	Forward	CTG​GCA​CAC​GGG​ACT​TGG​AAA​G	141	61.85
	Reverse	GCG​ATG​ACT​CAG​CAC​CTC​CAT​G		61.85
ROCK1	Forward	TGT​CCG​TGC​CTC​TCC​TCG​AAC	119	61.71
	Reverse	TCC​AAC​ACA​GCA​GTA​GGT​CAC​ATG		60.40
ROCK2	Forward	AGA​CAG​GGA​GGT​ACG​ACT​TGG​AAG	114	62.11
	Reverse	ACC​ACT​GGA​GCT​GCC​GTC​TC		61.55
NFATC3	Forward	TCC​ACA​AGG​CAT​TGA​GAC​ACA​TCC	94	60.40
	Reverse	CTC​ACC​AGC​AGC​AGC​AGC​AG		61.55
STAT3	Forward	GAA​CTG​AGT​GAG​CGT​GGG​TGA​TG	128	61.99
	Reverse	AGG​ACA​GGC​GGA​CAG​AAC​ATA​GG		61.99
β-Actin	Forward	TGT​CAC​CAA​CTG​GGA​CGA​TA	165	60.00
	Reverse	GGG​GTG​TTG​AAG​GTC​TCA​AA		60.00

### Western Blotting

The protein expressions of collagen 1, Bcl-2, Bax, caspase-3, ROCK1, ROCK2, NFAT_C_3, and P-STAT3 in the RV tissues were analyzed using western blotting. Snap-frozen RV tissues were homogenized, and protein concentration in the supernatant was determined using the BCA protein assay kit (Beyotime Institute of Biotechnology, Shanghai, China). The proteins (50 μg•lane^−1^) were separated using SDS-PAGE and transferred onto the polyvinyl difluoride membranes. The membranes were blocked with TBST containing 5% non-fat dry milk and incubated with anti-collagen 1 (1:2000), anti-P-STAT3 (1:2000), anti-Bcl-2 (1:1000), anti-Bax (1:2000), anti-ROCK1 (1:4000), anti-ROCK2 (1:1000), anti-NFATc3 (1:4000), anti-cleaved caspase-3 (1:1000), and anti-β-actin (1:5000) antibodies overnight at 4°C. The membranes were then incubated with secondary goat anti-mouse/rabbit IgG at a dilution of 1:5000 and subsequently visualized using the enhanced chemiluminescence (ECL) kit (Beyotime Institute of Biotechnology, Shanghai, China). β-actin was used as a loading control.

### Statistical Analysis

Data were analyzed using SPSS 18.0 software (SPSS, Inc., Chicago, IL, United States). Quantitative data are shown as the means ± standard deviation (SD). Dunnett’s test or the Student-Newman-Keuls test was used for multiple comparisons after one-way analysis of variance (ANOVA). A probability level of *p* ≤ 0.05 was considered significant. ANOVA was used to compare the means among different groups. *p* ≤ 0.05 was considered statistically significant.

## Results

### Tsantan Sumtang Restored RV Function in HPH Rats

**FIGURE 1:**
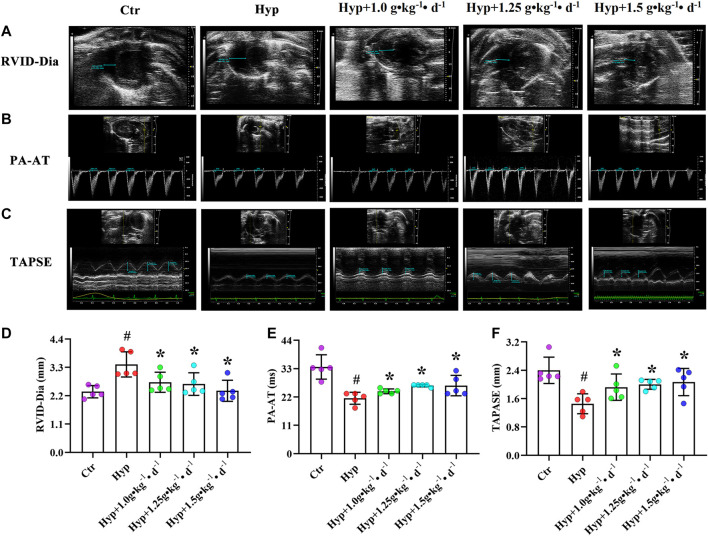
Tsantan Sumtang restored RV function in HPH rat. Rats were divided into following groups: control group (Ctr), hypoxia group (Hyp, rats were exposed under hypoxia in hypobaric chamber, equal to the parameter in altitude 4500 m for 4 weeks) and hypoxia (Hyp) + Tsantan Sumtang groups: rats were exposed under hypoxia in hypobaric chamber and treated by Tsantan Sumtang (1.0 g•kg-1•d-1, 1.25 g•kg-1•d-1, 1.5 g•kg-1•d-1) for 4 weeks. **(A and D)** Analysis of RV internal diameter during diastole (RVID-Dia) in HPH rat. **(B and E)** Pulmonary artery acceleration time (PA-AT) analysis in HPH rat. **(C and F)** Analysis of annular plane systolic excursion (TAPSE) in HPH rat. RVID-Dia, PA-AT and TAPSE were assessed by echocardiography after 4 weeks. Dotted blue lines in A to C highlight the detection distance. Data was expressed as means ±S.D. (^#^P<0.05 vs control, *P<0.05 vs hypoxia group).

To investigate whether Tsantan Sumtang improves RV function in HPH rats, we used noninvasive echocardiography. We found that RVID-Dia values increased under hypoxia. But after treating the rats with Tsantan Sumtang (1.0, 1.25, and 1.5 g•kg^−1^•d^−1^), the RVID-Dia values significantly decreased (*p* < 0.05 vs. hypoxia group, Figures 1A and 1D). The PA-AT values decreased under hypoxia and significantly increased in Tsantan Sumtang (1.25 g•kg^−1^•d^−1^) and Tsantan Sumtang (1.5 g•kg^−1^•d^−1^) treatment groups (*p* < 0.05 vs. hypoxia group, Figures 1B and 1E). Similarly, TAPSE also decreased under hypoxia and significantly increased after Tsantan Sumtang treatment (*p* < 0.05 vs. hypoxia group, Figures 1C and 1F).

### Tsantan Sumtang Improved RV-PA Coupling in HPH Rats

RV function is critical to PAH, and the relationship between RV contractility and pulmonary artery load is often referred to as RV-PA coupling (Aggarwal et al., 2018). The end-systolic/arterial elastance (Ees/Ea) ratio from invasive pressure-volume loops was used to assess the RV-PA coupling. Other parameters, including FAC (%) and the TAPSE/PASP, FAC/mPAP, and PASP/ESA ratios, were also found to be effective evaluation indexes for RV-PA coupling assessment (Tello et al., 2019b). In this study, FAC (%) and TAPSE/PA-AT, TAPSE/PASP, and FAC/mPAP ratios were significantly decreased in the hypoxia group, indicating that RV-PA coupling changed under hypoxia. However, these indexes significantly increased after the rats were treated with Tsantan Sumtang (*p* < 0.05 vs. hypoxia group, Table 2).

**TABLE 2:** Tsantan Sumtang treatment improved RV-arterial coupling in HPH rat $\left(\bar{x}\pm s\right)$.

Parameters	Number of rats	Ctr	Hyp	Hyp + 1.0 g kg^−1^•d^−1^	Hyp + 1.25 g kg^−1^•d^−1^	Hyp + 1.5 g kg^−1^•d^−1^
TAPSE/PASP (mm•mmHg^−1^)	5	0.7272 ± 0.01	0.0332 ± 0.01^a^	0.0552 ± 0.01^b^	0.0623 ± 0.01^b^	0.0803 ± 0.02^b^
FAC (%)	5	41.0540 ± 6.65	26.9523 ± 6.39^a^	41.1126 ± 14.79^b^	41.5595 ± 11.13^b^	43.7362 ± 4.43^b^
FAC/mPAP (%•mmHg^−1^)	5	2.7268 ± 0.13	0.8972 ± 0.26^a^	1.5659 ± 0.40^b^	1.6624 ± 0.50^b^	2.0248 ± 0.63^b^
TAPSE/PA-AT (mm•ms^−1^)	5	0.0713 ± 0.01	0.0677 ± 0.02^a^	0.0795 ± 0.02^b^	0.0758 ± 0.01^b^	0.0783 ± 0.02^b^

FAC (%): fractional area change, calculated as (end-diastolic area−ESA)/ESA*100% (ESA represented end-systolic area). mPAP: mean pulmonary artery pressure (assessed by right heart catheterization). PA-AT: pulmonary artery acceleration time, obtained from echocardiography. PASP: systolic pulmonary artery pressure. TAPSE: tricuspid annular plane systolic excursion, obtained from echocardiography. Results were expressed as mean ± S.D. Rats were divided into the following groups: control group (Ctr), hypoxia group (Hyp, rats were exposed under hypoxia in hypobaric chamber, equal to the parameter in altitude 4500 m for 4 weeks), and hypoxia (Hyp) + Tsantan Sumtang groups: rats were exposed under hypoxia in hypobaric chamber and treated by Tsantan Sumtang (1.0, 1.25, 1.5 g•kg^−1^•d^−1^) for 4 weeks.

^a^ *p* < 0.05 vs. control group.

^b^ *p* < 0.05 vs. hypoxia group.

### Tsantan Sumtang Recovered Hemodynamic and Hematological Index in HPH Rats

**FIGURE 2:**
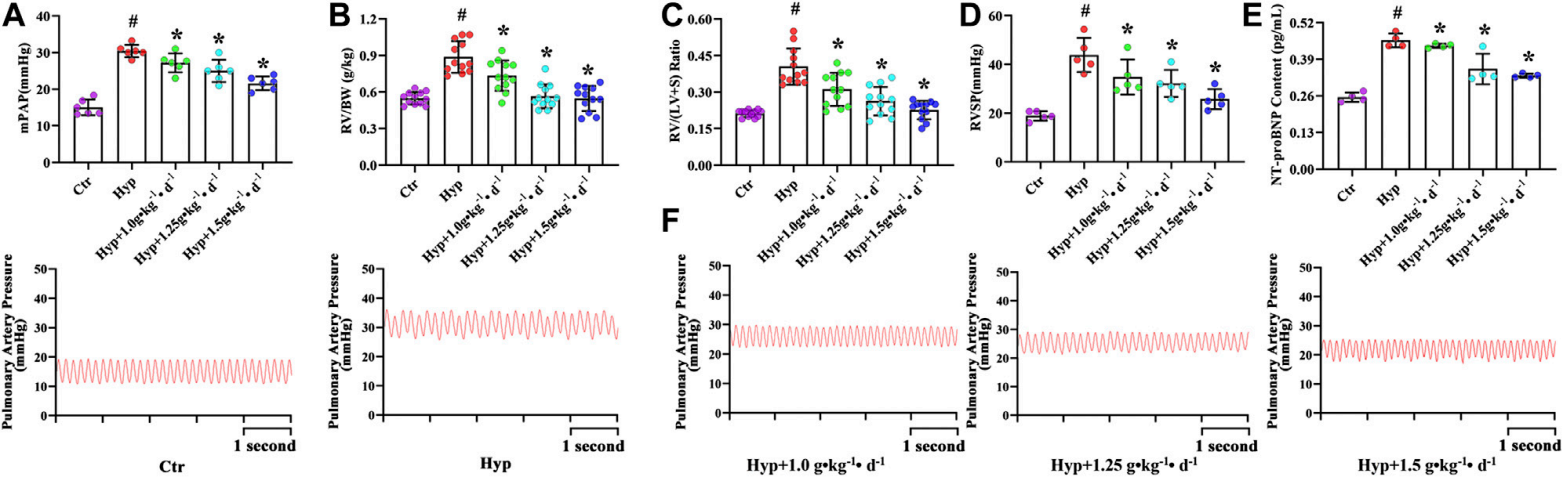
Effect of Tsantan Sumtang on hematological indexes in HPH rat. **(A)** mPAP, indicated mean pulmonary arterial pressure, **(B)** RV/BW, indicated the ratio of RV to body weight (BW), **(C)** RV/LV + S, indicated the ratio of RV weight to left ventricle (LV) with septum (S) weight, **(D)** RVSP, indicated RV systolic pressure. **(E)** NT-proBNP content, indicated the content of NT-proBNP in blood in HPH rat. **(F)** Pictures of mPAP wave in each group. Data was expressed as means ±S.D. (^#^P<0.05 vs control group, *P<0.05 vs hypoxia group).

Hypoxic rats were found to be lethargic, and their body weight increased from 160.19 ± 6.38 to 216.99 ± 18.22 g (Table 3). The RV/BW ratio was significantly higher in the hypoxia group as compared with that in the control group (*p* < 0.05, Figure 2B). However, after Tsantan Sumtang treatment (1.0, 1.25, and 1.5 g•kg^−1^•d^−1^), the rats showed a significant decrease in the RV/BW ratio (*p* < 0.05, vs. HPH group, Figure 2B). The mPAP and RVSP values were also higher in the hypoxia group as compared with the corresponding values in the control group. After Tsantan Sumtang treatment (1.0, 1.25, and 1.5 g•kg^−1^•d^−1^), the levels of mPAP and RVSP were decreased as compared with the relative levels in the hypoxia group (without Tsantan Sumtang treatment) (*p* < 0.05, Figures 2A,D,F). Further, the RV/LV + S ratio was significantly higher in the hypoxia group as compared with that in the control group (*p* < 0.05, Figure 2C). The RV/LV + S ratios were significantly lower in Tsantan Sumtang (1.25 and 1.50 g•kg^−1^•d^−1^) groups as compared with those in the hypoxia group (*p* < 0.05, Figure 2C).

**TABLE 3:** Effect of Tsantan Sumtang treatment on anatomic data in HPH rat $\left(\bar{x}\pm s\right)$.

Parameters	Ctr	Hyp	Hyp + 1.0 g•kg^−1^•d^−1^	Hyp + 1.25 g•kg^−1^•d^−1^	Hyp + 1.5 g•kg^−1^•d^−1^
Number of rats	11	12	11	12	12
Body weight	195.89 ± 16.33	216.99 ± 18.30	228.98 ± 17.74	233.29 ± 35.57	221.12 ± 24.46
Ratio of liver weight to body weight (mg•kg^−1^)	49.33 ± 6.43	31.27 ± 8.20	32.84 ± 3.41	30.53 ± 1.30	32.28 ± 1.41
Ratio of spleen weight to body weight (mg•kg^−1^)	2.46 ± 0.69	1.70 ± 0.40	1.75 ± 0.30	1.99 ± 0.56	1.89 ± 0.33
Ratio of lung weight to body weight (mg•kg^−1^)	6.16 ± 0.83	7.38 ± 0.77	7.15 ± 0.73	6.59 ± 1.43	6.36 ± 1.21
Ratio of kidney weight to body weight (mg•kg^−1^)	9.57 ± 0.81	7.56 ± 0.66	7.49 ± 0.51	7.48 ± 0.94	7.84 ± 0.54

Results were expressed as mean + SD. These data did not show significant difference in body weight and organ coefficients of rats among hypoxia group and Tsantan Sumtang treatment groups.

RBC count, HGB, and Hct values were significantly higher, while WBC and PLT counts were significantly lower in the rats with four weeks of exposure to hypoxia, similar to the findings of Harrington et al. (Harrington et al., 2010). However, Tsantan Sumtang treatment restored the changes in RBC, HGB, Hct, WBC, and PLT values (*p* < 0.05, Table 4). As compared with the control group rats, the hypoxia group rats had significantly higher levels of NT-proBNP, and the levels decreased after treating the rats with Tsantan Sumtang (1.0, 1.25, and 1.5 g•kg^−1^•d^−1^, *p* < 0.05, Figure 2E). However, there were no significant differences in body weight and organ coefficients among these groups (*p* < 0.05, Table 3).

**TABLE 4:** Effect of Tsantan Sumtang treatment on hematological index in HPH rat $\left(\bar{x}\pm s\right)$.

Group	HGB (g•L^−1^)	Hct (%)	RBC (10^12^•L^−1^)	WBC (10^9^•L^−1^)	PLT (10^9^•L^−1^)
Number of rats	11	12	11	12	12
Ctr	171.6 ± 5.17	54.14 ± 3.32	7.2 ± 0.82	7.36 ± 0.48	679.2 ± 66.84
Hyp	233.8 ± 4.49^a^	66.56 ± 1.48^a^	11.39 ± 0.53^a^	4.45 ± 0.65^a^	589.8 ± 31.27^a^
Hyp + 1 g•kg^−1^•d^−1^	224.8 ± 16.16^b^	64.48 ± 1.98	9 ± 0.90^b^	5.22 ± 2.33	627.4 ± 45.74^b^
Hyp + 1.25 g•kg^−1^•d^−1^	205.8 ± 12.15^b^	63.98 ± 1.24^b^	10.25 ± 0.38^b^	5.8 ± 0.99	626.2 ± 37.61
Hyp + 1.5 g•kg^−1^•d^−1^	183.0 ± 7.43^b^	57.62 ± 2.21^b^	10.15 ± 0.55^b^	6.81 ± 1.01^b^	632.6 ± 30.58^b^

HGB: hemoglobin, Hct: hematocrit, RBC: red blood cell, WBC: white blood cell, and PLT: platelet. Results were expressed as mean ± S.D.

^a^ *p* < 0.05 vs. control group.

^b^ *p* < 0.05 vs. hypoxic group.

### Tsantan Sumtang Protected RV Against Structural Maladaptive Remodeling

**FIGURE 3:**
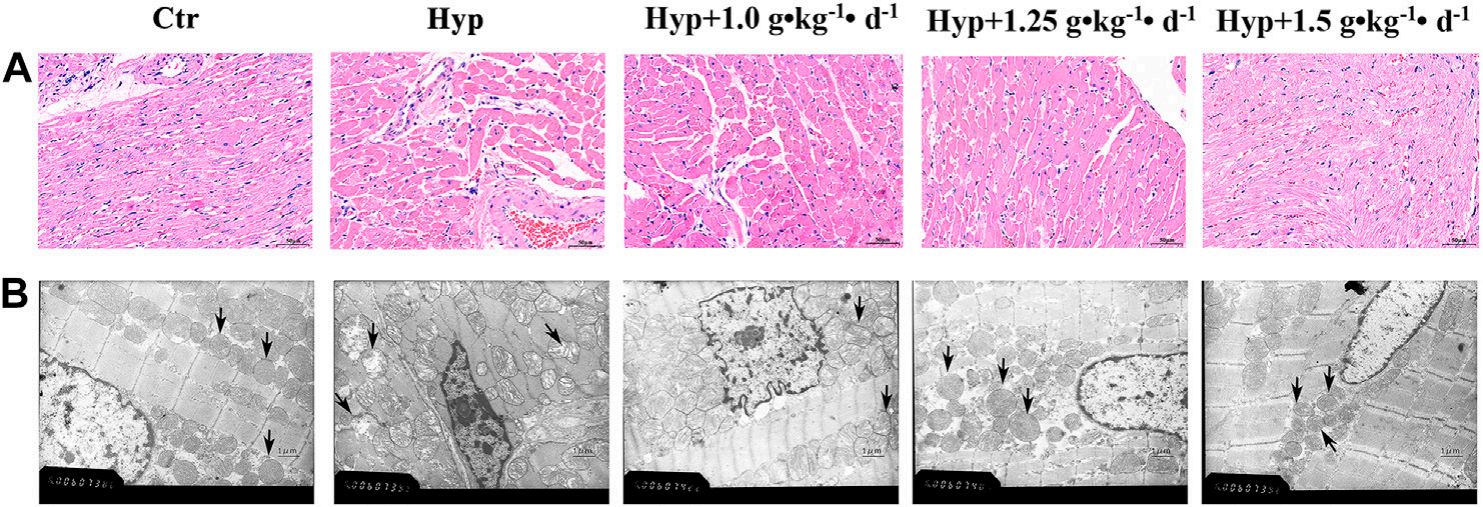
Effect of Tsantan Sumtang on morphological changes of RV tissue in HPH rat. **(A)** Representative photomicrograph of the morphological changes of RV tissue in HPH rat by HE staining (400 ×). **(B)** Representative photomicrographs of the ultrastructure observed by transmission electron microscope (TEM, × 20000). Black arrow indicated the mitochondria changes in each group. n=4.

Using H&E staining, we found markedly hypertrophied and sparsely distributed myocardial fibers in the RV tissues. Moreover, some fibers in the hypoxia group were found to be dissolved and hence were not evident as compared with the control group. Tsantan Sumtang treatments (1.0, 1.25, and 1.5 g•kg^−1^•d^−1^) significantly alleviated the myocardial tissue changes (Figure 3A). Changes in the ultrastructure of cardiac tissues were observed using TEM. TEM images of the control group showed evenly distributed chromatin of cardiomyocytes and abundant mitochondria in the cytoplasm with a clear structure and neat arrangement of muscle fibers. However, in the hypoxia group, mitochondria swelling was observed, and the mitochondrial structures were not entirely evident. Mitochondrial ultrastructural injuries were significantly attenuated in the Tsantan Sumtang treatment groups (1.0, 1.25, and 1.5 g•kg^−1^•d^−1^, Figure 3B).

**FIGURE 4:**
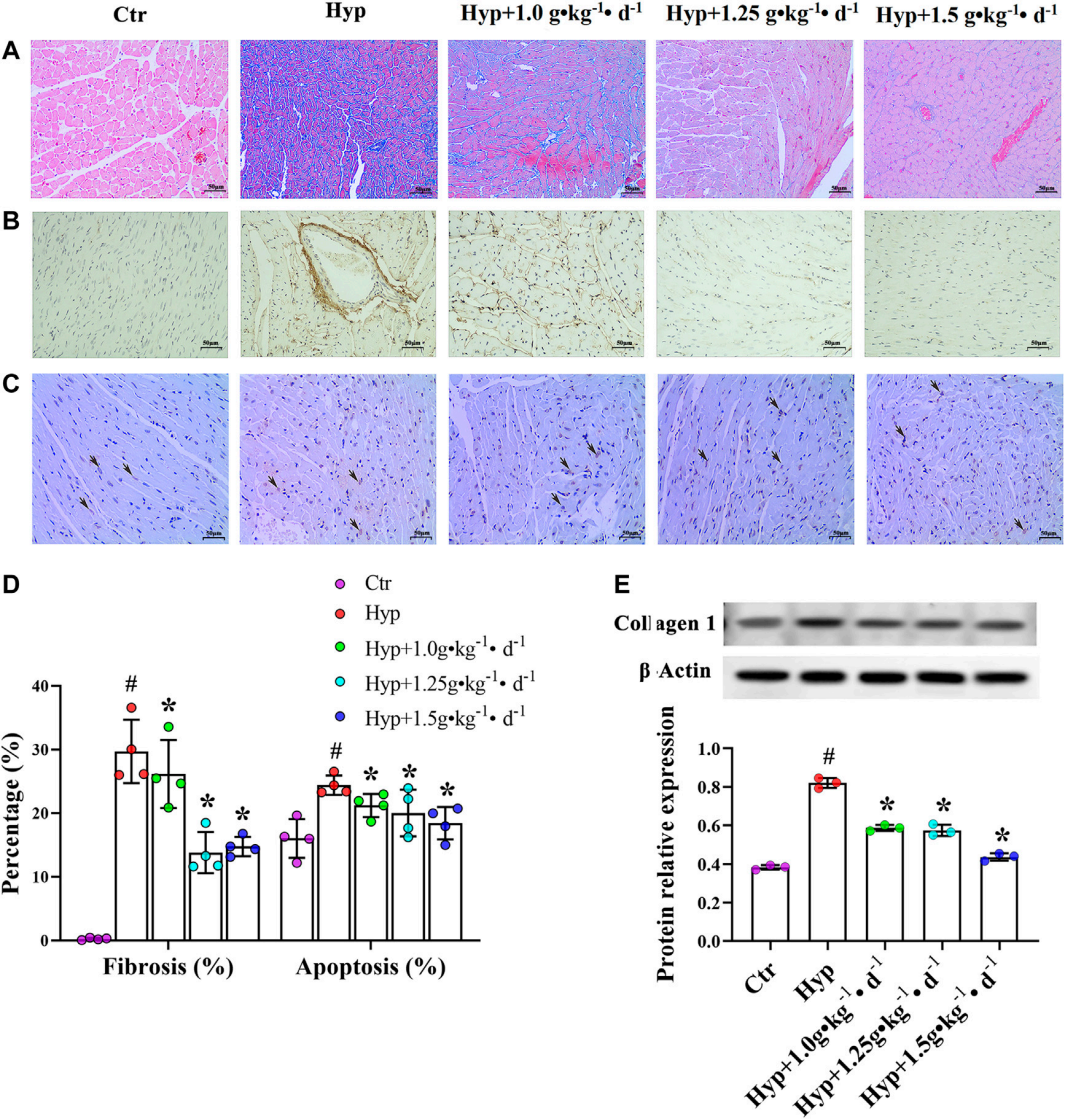
Effect of Tsantan Sumtang on RV structure in HPH rat. (A) Fibrosis analysis of RV tissue, evaluated by Masson's trichrome staining (400 ×). (B) Collagen 1 expression level detected by immunohistochemical (400 ×). (C) Apoptosis analysis of RV tissue, assessed by TUNEL assay (black arrows point to positive spots, 400 ×). (D) Statistical analysis of fibrosis and apoptosis of RV tissue. (E) Analysis of collagen 1 expression level, detected by western blotting. Data was expressed as means±S.D. (^#^P<0.05 vs control group, *P<0.05 vs hypoxia group).

Masson’s trichrome stain was used to evaluate the effect of Tsantan Sumtang on RV fibrosis. Immunohistochemical methods and western blotting were used to detect the expression of collagen 1. Fibrosis was apparent in RV of the hypoxia group rats, as illustrated by the increased collagen-specific blue color in Masson’s trichrome-stained tissue (Figures 4A,D) and the increased expression of collagen 1 (Figures 4B,E). Tsantan Sumtang treatments (1.0, 1.25, and 1.5 g•kg^−1^•d^−1^) markedly reduced the overall collagen content and collagen 1 expression levels in RV tissues. In order to study apoptosis-induced cardiomyocyte death, we used the TUNEL assay and examined the degree of RV apoptosis. RV tissues of the hypoxia group rats exhibited an increase in the rate of apoptosis as compared with that of the control group rats. The rate of apoptosis was lower in the Tsantan Sumtang treated groups (1.0, 1.25, and 1.5 g•kg^−1^•d^−1^, Figures 4C,D).

### Antioxidant Activity of Tsantan Sumtang Improved RV Function

**FIGURE 5:**
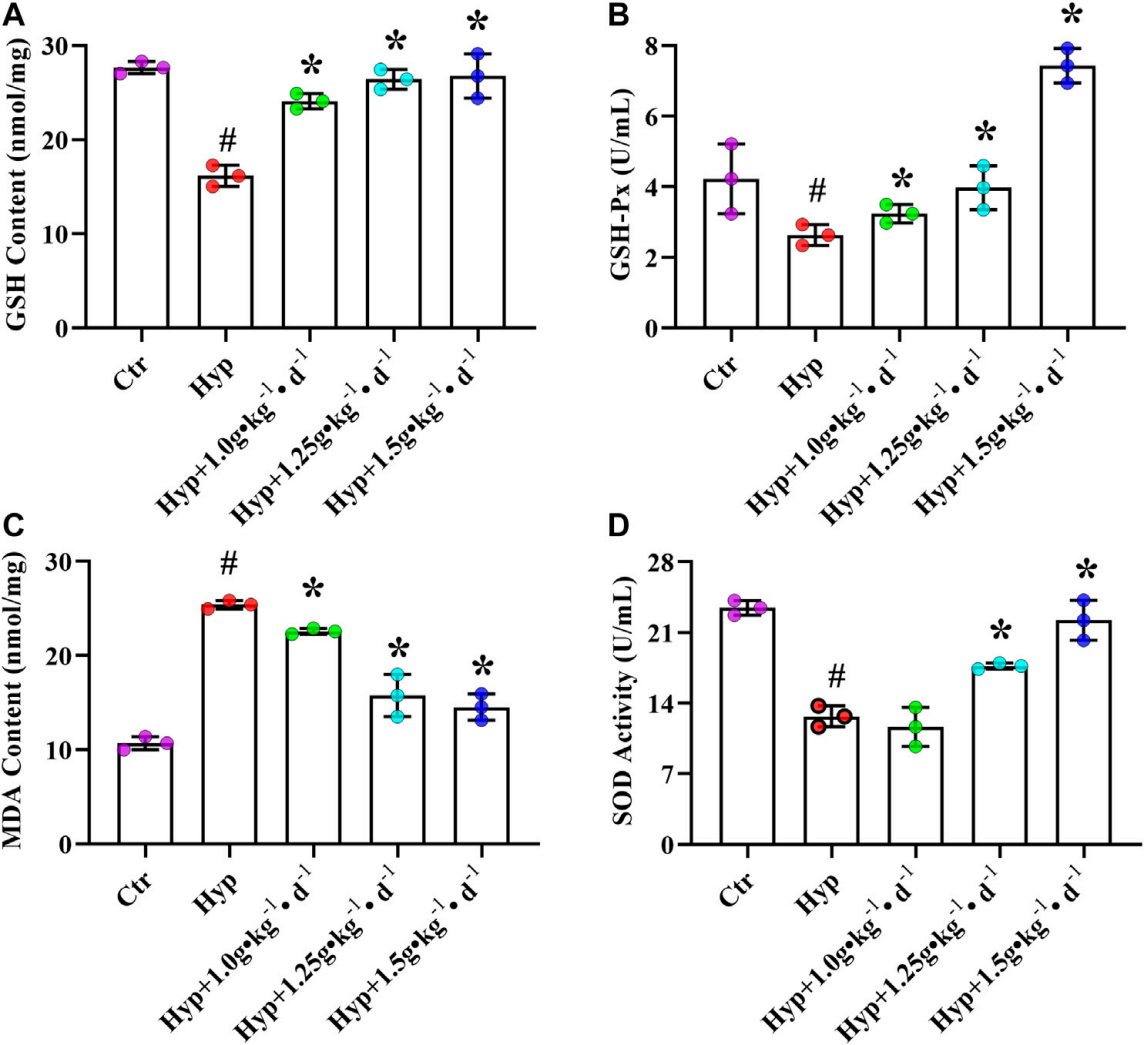
Effect of Tsantan Sumtang on oxidant stress of RV tissue in HPH rat. **(A)** GSH content analysis of RV tissue. **(B)** GSH-Px activity analysis of RV tissue. **(C)** MDA content analysis of RV tissue. **(D)** SOD activity analysis of RV tissue. Data was expressed as means ± S.D. (^#^P<0.05 vs control group, *P<0.05 vs hypoxia group).

Except for RV fibrosis and apoptosis-induced RV decompensation and maladaptive remodeling, other factors that influence the RV function include oxidative stress (He et al., 2016). We observed a substantial increase in MDA content and a significant decrease in GSH-PX activity, GSH content, and SOD activity in the RV tissues of the hypoxia group rats (*p* < 0.05, Figure 5). After treating the rats with Tsantan Sumtang (1.0, 1.25, and 1.5 g•kg^−1^•d^−1^), we observed a significant increase in GSH content and GSH-PX activity. Moreover, the MDA content was lower, and the SOD activity was higher as compared with the corresponding values in the hypoxia group (*p* < 0.05, Figures 5A–D).

The pharmacological mechanism of Tsantan Sumtang involved regulation of ROCK signaling pathway and its downstream effectors.

**FIGURE 6:**
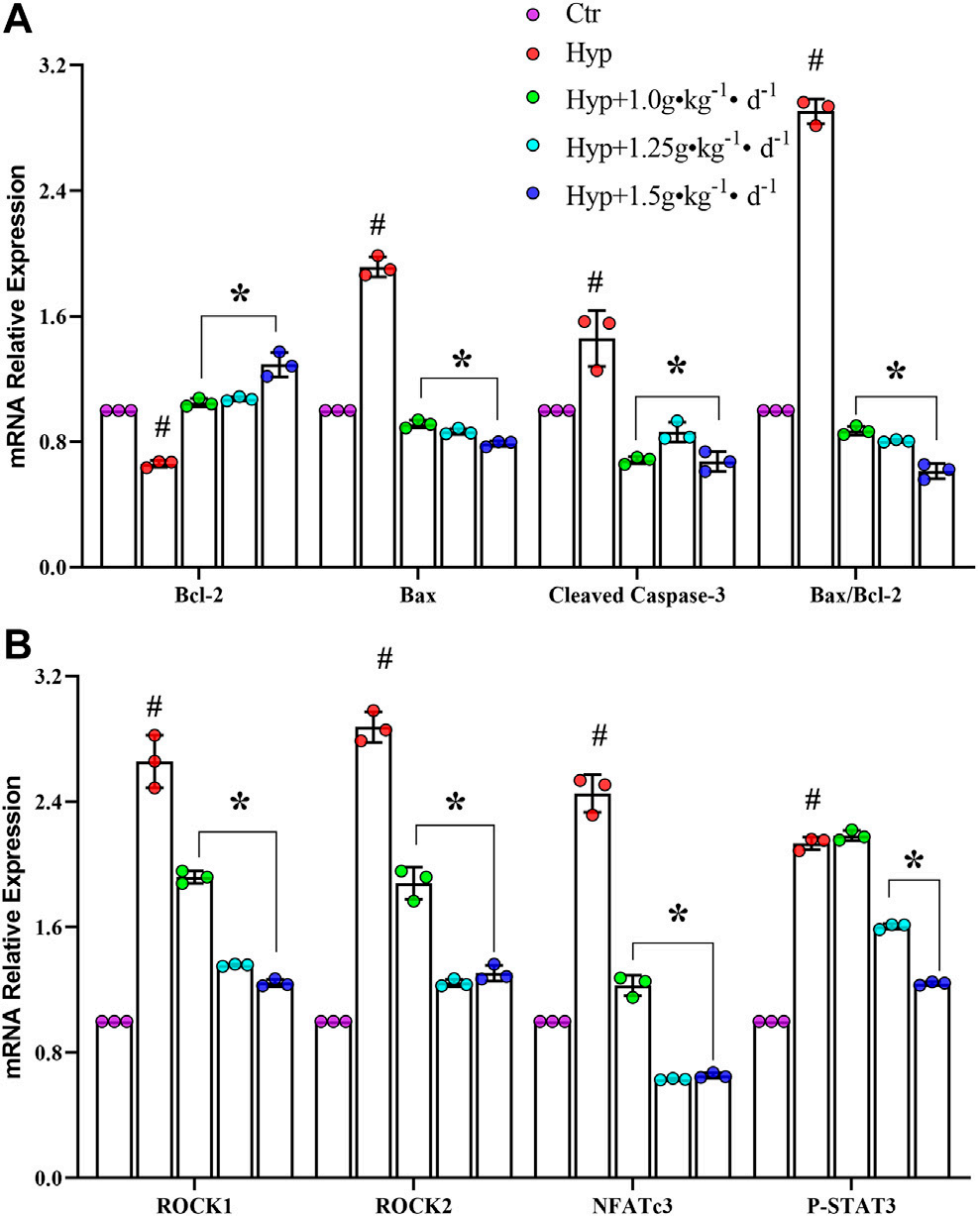
Effect of Tsantan Sumtang on Bcl-2, Bax, cleaved caspase-3, ROCK1, ROCK2, NFATc3 and STAT3 mRNA expression of RV tissue in HPH rat. **(A)** Statistical analysis of Bcl-2, Bax and cleaved caspase-3 mRNA expression level of RV tissue. **(B)** Statistical analysis of ROCK1, ROCK2, NFATc3 and STAT3 mRNA expression level in RV tissue. Data are mean±S.D. of three identical experiments (^#^P<0.05 vs control group, *P<0.05 vs hypoxia group).

**FIGURE 7:**
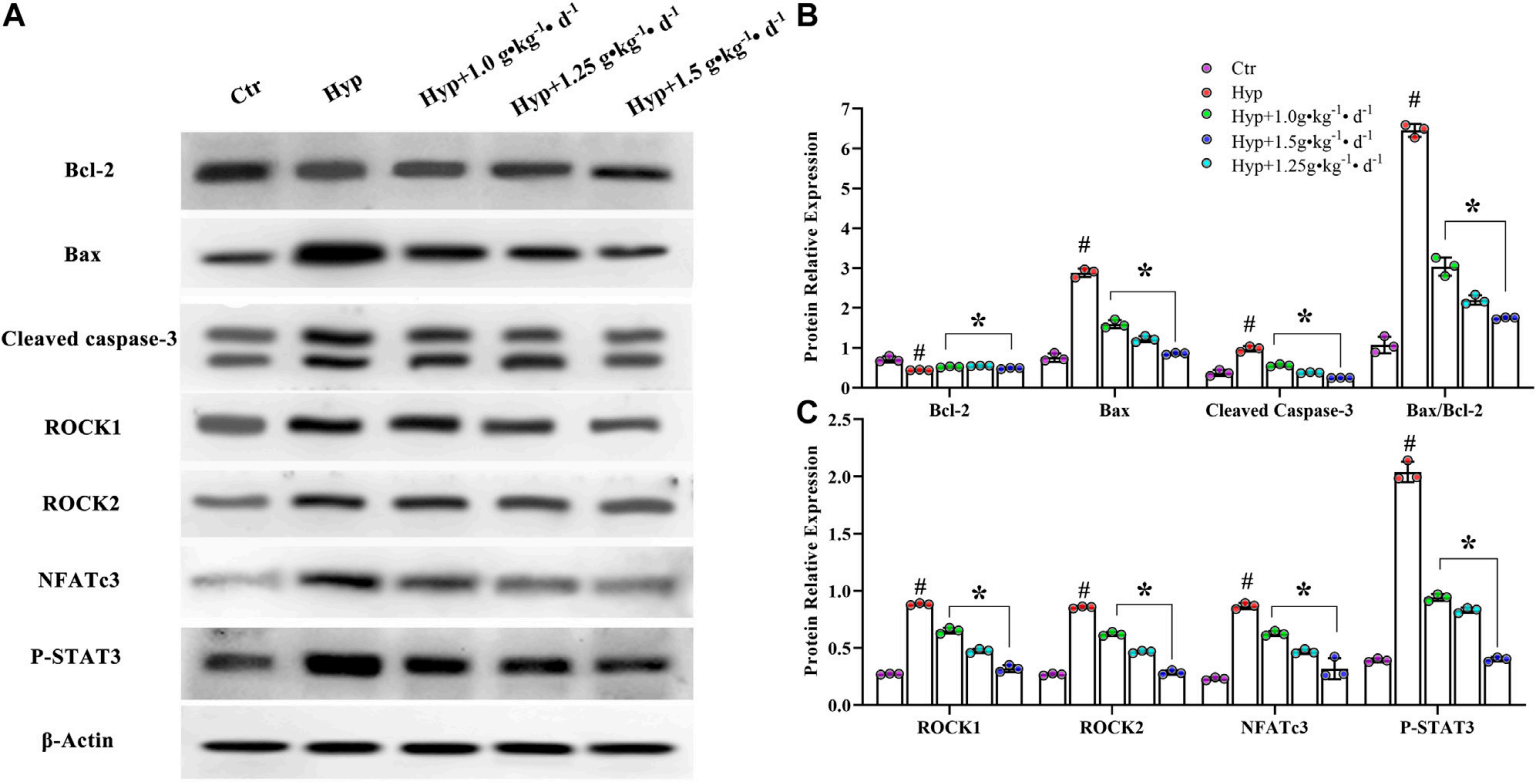
Effect of Tsantan Sumtang on Bcl-2, Bax, cleaved caspase-3, ROCK1, ROCK2, NFATc3 and SATT3 protein expression of RV tissue in HPH rat. **(A)** Protein bands of Bcl-2, Bax, cleaved caspase-3, ROCK1, ROCK2, NFATc3 and STAT3. **(B)** Statistical analysis of Bcl-2, Bax and cleaved caspase-3 expression level of RV tissue. **(C)** Statistical analysis of ROCK1, ROCK2, NFATc3 and P-STAT3 expression level in RV tissue. Data was expressed as mean ± S.D. (^#^P<0.05 vs control group, *P<0.05 vs hypoxia group).

To understand how Tsantan Sumtang restores the RV function, we studied the effect of this medicine on the ROCK signaling pathway. The ROCK pathway is reported to be hyperactivated in various cardiovascular diseases (Wang et al., 2019). The mRNA and protein levels of Bax and cleaved caspase-3 were significantly lower, while the corresponding levels of Bcl-2 were significantly higher in the Tsantan Sumtang groups (1.0, 1.25, and 1.5 g•kg^−1^•d^−1^) as compared with the hypoxia group (*p* < 0.05, Figures 6A, 7A,B). The Bax/Bcl-2 ratio is reported to be more critical for apoptosis than the concentration of either protein. A higher Bax/Bcl-2 ratio upregulates caspase-3, resulting in an increase in apoptosis. We found a significantly higher Bax/Bcl-2 ratio in the hypoxia group as compared with the control group. However, the ratio significantly decreased when the rats were treated by Tsantan Sumtang (1.0, 1.25, and 1.5 g•kg^−1^•d^−1^, *p* < 0.05 vs. hypoxia group, Figures 6A, 7A,B). The mRNA and protein expression levels of ROCK1 and ROCK2 were also significantly lower in the RV tissues of Tsantan Sumtang treated rats as compared with those of hypoxia group rats (*p* < 0.05, Figures 6B, 7A,C). The inhibition of ROCK activity was accompanied by a decrease in the active dephosphorylated form of the nuclear factor of activated T cells, cytoplasmic 3 (NFATc3), and the active phosphorylated form of STAT3 (*p* < 0.05, Figures 6B, 7A,C).

### Gas Chromatography-Mass Spectrometry of the Essential Oil of Tsantan Sumtang

**FIGURE 8:**
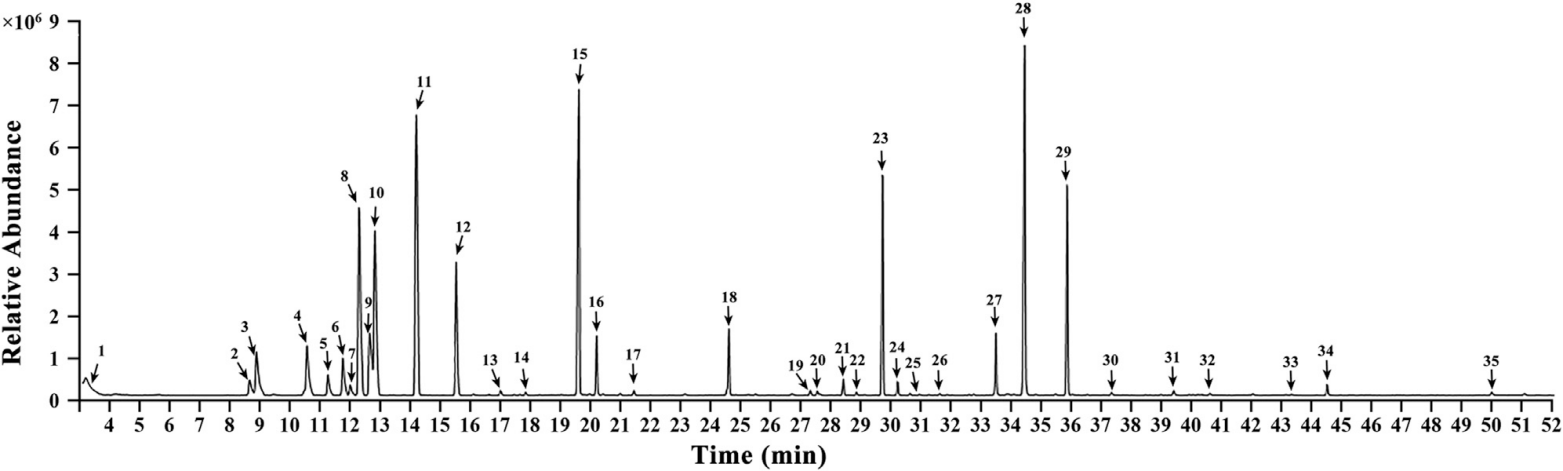
Gas chromatographymass spectrometer analysis chromatogram in volatile oil of Tsantan Sumtang.

The constituent profiling of the aqueous extract of Tsantan Sumtang was carried out as our previously published procedures (Dang et al., 2020). In this study, we identified 35 components in the essential oil of Tsantan Sumtang using GC-MS. The identification percentage of the constituents was 98.84%, and information about the retention time, relative content, retention index (TI), and identification was listed in Figure 8 and Table 5. Out of 35 identified components, 16 components had more than 1% relative content. For instance, the contents of γ-Terpinene, 1,3-Benzodioxole, 4-methoxy-6-(2-propenyl)-, and Terpinen-4-ol were more than 10%. These components are also the main constituents of the essential oil of *Myristica fragrans* Houtt. Our findings are similar to those of Ma et al. (Ma et al., 2018). Moreover, β-Santalol, α-Bisabolol, α-Bisabolene, and α-Bergamotene from *Santalum album* L. were also identified in the essential oil of Tsantan Sumtang.

**TABLE 5:** Chemical composition of volatile oil from Tsantan Sumtang. T_R_: retention time. TI: retention index.

No.	T_R_ (min)	Relative content (%)	TI	Identification	Cas	Molecular formula
1	3.22	2.76	-	Furfuryl alcohol	16015-08-0	C_6_H_12_O_2_
2	8.67	0.85	-	α-Pinene	80-56-8	C_10_H_16_
3	8.90	2.81	-	(1R)-2,6,6-Trimethylbicyclo[3.1.1]hept-2-ene	7785-70-8	C_10_H_16_
4	10.58	2.87	-	β-Phellandrene	555-10-2	C_10_H_16_
5	11.28	0.96	-	Bicyclo[3.1.0]hexane, 4-methylene-1-(1-methylethyl)-	3387-41-5	C_10_H_16_
6	11.78	1.80	1001	α-Phellandrene	99-83-2	C_10_H_16_
7	12.03	0.47	1008	3-Carene	13466-78-9	C_10_H_16_
8	12.32	9.75	1015	(+)-4-Carene	29050-33-7	C_10_H_16_
9	12.68	3.65	1023	*o*-Cymene	527-84-4	C_10_H_14_
10	12.85	8.30	1026	Cyclohexene, 4-methylene-1-(1-methylethyl)-	99-84-3	C_10_H_16_
11	14.22	14.18	1057	γ-Terpinene	99-85-4	C_10_H_16_
12	15.55	5.21	1087	Cyclohexane, 1-methylene-4-(1-methylethenyl)-	499-97-8	C_10_H_16_
13	17.03	0.17	1119	Bicyclo[3.1.0]hexan-2-ol, 2-methyl-5-(1-methylethyl)-, (1α,2α,5α)-	17699-16-0	C_10_H_18_O
14	17.87	0.11	1137	2-Cyclohexen-1-ol, 1-methyl-4-(1-methylethyl)-, *trans*-	29803-81-4	C_10_H_18_O
15	19.64	11.59	1176	Terpinen-4-ol	562-74-3	C_10_H_18_O
16	20.23	1.70	1188	α-Terpineol	98-55-5	C_10_H_18_O
17	21.47	0.13	1216	2-Cyclohexen-1-ol, 1-methyl-4-(1-methylethyl)-, *cis*-	29803-82-5	C_10_H_18_O
18	24.63	1.98	1286	Safrole	94-59-7	C_10_H_10_O_2_
19	27.35	0.14	1349	Copaene	3856-25-5	C_15_H_24_
20	27.59	0.12	1354	Cyclopentane, 1-methyl-3-(2-methyl-1-propenyl)-	75873-01-7	C_10_H_18_
21	28.45	0.44	1374	α-Cubebene	17699-14-8	C_15_H_24_
22	28.89	0.08	1384	Bicyclo[3.1.1]heptane, 6,6-dimethyl-2-methylene-, (1S)-	18172-67-3	C_10_H_16_
23	29.75	6.82	1405	Methyleugenol	93-15-2	C_11_H_14_O_2_
24	30.25	0.40	1417	Caryophyllene	87-44-5	C_15_H_24_
25	30.98	0.03	1435	α-Bergamotene	18252-46-5	C_15_H_24_
26	31.66	0.05	1451	α-Bisabolene	29837-07-8	C_15_H_24_
27	33.52	1.72	1497	Benzene, 1,2-dimethoxy-4-(1-propenyl)-	93-16-3	C_11_H_14_O_2_
28	34.48	12.48	1522	1,3-Benzodioxole, 4-methoxy-6-(2-propenyl)-	607-91-0	C_11_H_12_O_3_
29	35.89	6.60	1558	Benzene, 1,2,3-trimethoxy-5-(2-propenyl)-	487-11-6	C_12_H_16_O_3_
30	37.38	0.08	1596	Guaiol	489-86-1	C_15_H_26_O
31	39.45	0.14	1652	Isoelemicin	487-12-7	C_12_H_16_O_3_
32	40.65	0.05	1685	α-Bisabolol	515-69-5	C_15_H_26_O
33	43.36	0.03	1761	β-Santalol	77-42-9	C_15_H_24_O
34	44.56	0.29	1795	Tetradecanoic acid, ethyl ester	124-06-1	C_16_H_32_O_2_
35	50.04	0.09	1961	Dibutyl phthalate	84-74-2	C_16_H_22_O_4_

## Discussion

PAH is not a single disease but a combination of severe cardiac and pulmonary complications caused by diverse factors, including hypoxia (Liu et al., 2019). RV function was thought to be the most reliable indicator of prognosis in PAH patients (Kawakubo et al., 2019). Tsantan Sumtang, as one of the medicines of the traditional Tibetan medicine, serves to decrease mPAP and alleviates RV remodeling and fibrosis in HPH rats (He et al., 2018; Dang et al., 2020). In this study, we investigated the effect of Tsantan Sumtang on the RV function in HPH rats and studied the underlying mechanism. We used echocardiography to assess the RV function. The values of TAPSE, a critical index for the prediction of RV ejection and systolic function (Tello et al., 2019b), significantly decreased under hypoxia. These values significantly increased after treating the rats with Tsantan Sumtang, demonstrating the protective effect of this medicine on RV ejection and systolic function (*p* < 0.05 vs. hypoxia group, Figures 1C,F). Moreover, RVID-Dia was increased under hypoxia. As compared with the RVID-Dia of the hypoxia group, RVID-Dia of the Tsantan Sumtang group was lower (*p* < 0.05, Figures 1A,D). PA-AT was significantly lower in the hypoxia group as compared with the control group, and PA-AT in the Tsantan Sumtang group was significantly higher than that in the hypoxia group (*p* < 0.05, Figures 2B,E). NT-proBNP, secreted by cardiomyocytes, is an established noninvasive marker for RV function (Patel et al., 2020). NT-proBNP is also an independent predictor of survival in PAH patients. NT-proBNP level was increased under hypoxia. It was found to be significantly lower after Tsantan Sumtang treatment (*p* < 0.05 vs. hypoxia group, Figure 2E).

RV-PA coupling represents the relationship between RV contractility and RV afterload (Aggarwal et al., 2018). RV-PA coupling is an independent predictor of RV function (Bellofiore and Chesler, 2013). Optimal RV-PA coupling maintains cardiac output and maximizes the work efficiency of the right heart (Aggarwal et al., 2018). As PAH progresses, the right ventricle adapts to the increasing afterload by enhancing the contractility to maintain the blood flow (“coupling”) (Tello et al., 2019b). However, RV-PA coupling progressively decreases in PAH patients. The gold standard of RV-PA coupling assessment is the evaluation of end-systolic/arterial elastance (Ees/Ea) ratio from invasive pressure-volume loops (Vonk Noordegraaf et al., 2017). This approach is technically demanding and expensive. At present, TAPSE/PASP ratio is regarded as a noninvasive surrogate for Ees/Ea ratio evaluation method (Tello et al., 2019b). TAPSE/PASP ratio is thought to be an independent predictor of PAH survival (Tello et al., 2019b). Other indicators, such as FAC (%) as well as FAC/mPAP and TAPSE/PA-AT ratio, are also reported to be the surrogates of Ees/Ea ratio evaluation for RV-PA coupling assessment (Tello et al., 2019b). Therefore, all these parameters, including TAPSE/PASP, FAC (%), FAC/mPAP, and TAPSE/PA-AT, could be used to evaluate the effect of Tsantan Sumtang on RV-PA coupling. We found that TAPSE/PASP, FAC (%), FAC/mPAP, and TAPSE/PA-AT were decreased under hypoxia and significantly increased after Tsantan Sumtang treatment (*p* < 0.05 vs. hypoxia group, Table 2).

We observed a significant increase in the major physiological parameters, including mPAP, RVSP, RV/BW, and RVHI, in rats maintained under hypoxia for 4 weeks (*p* < 0.05 vs. control group, Figures 2A–F), demonstrating that the rat model of HPH was successfully established. Meanwhile, the levels of Hct, HGB, and RBC were also higher, and those of WBC and PLT were lower in hypoxic rats (*p* < 0.05 vs. control group, Table 4). The treatment of Tsantan Sumtang led to a significant and dose-dependent decrease in mPAP, RVSP, RVHI, and RV/BW (*p* < 0.05 vs. hypoxia group, Figures 2A–F). Further, the levels of Hct, HGB, and RBC decreased, while those of WBC and PLT increased after Tsantan Sumtang treatment (*p* < 0.05 vs. hypoxia group, Table 4). Interestingly, in our previous study, the aqueous extract of Tsantan Sumtang was found to decrease the mPAP, RVSP, RVHI, and RV/BW levels in HPH rats without any significant dose-dependency. Meanwhile, the levels of WBC, HGB, Hct, and PLT did not significantly change after the treatment with the aqueous extract of Tsantan Sumtang (Dang et al., 2020). These variations might be due to the differences between the components of the powder (including essential oil) and that of the aqueous extract of Tsantan Sumtang (essential oil was excluded during decoction).

Cardiac fibrosis, a common occurrence in various pathogenic processes, is associated with the contractile and diastolic dysfunctions (Aguero et al., 2014). During diastole, myocardial collagen is essential for myocardium relaxation and filling of the ventricles; however, excessive fibrosis results in diastolic dysfunction. Meanwhile, collagen is essential for available force transmission during systole, but excessive collagen deposition might cause excitation-contraction coupling disturbances and impaired myocardial contraction (Andersen et al., 2019). Myocardial collagen deposition is regarded as the major factor involved in cardiac fibrosis; fibrosis is typically quantified via histological methods (Grignola et al., 2007; Li et al., 2007). Hypoxia is considered as one of the significant factors that induce cardiac fibrosis. We observed an increase in the fibrosis level in RV of the rats under hypoxia. After Tsantan Sumtang treatment, the fibrosis level in the rats was found to be significantly decreased (*p* < 0.05, vs. hypoxia group, Figures 4A,D). These findings were consistent with those of our previous study, which demonstrated that Tsantan Sumtang attenuates chronic hypoxia-induced right ventricular structure remodeling and fibrosis. Therefore, we assumed that the protective effect of Tsantan Sumtang on the RV function might be related to its protective effect on the RV structure.

The disruption of the redox homeostasis is associated with the pathogenesis of PAH. Excessive oxidative stress results in the activation of inflammatory signaling, proliferation, apoptosis-resistance, and hypertrophy of pulmonary arterial muscle cells, elevating pulmonary arterial pressure (Dos Santos Lacerda et al., 2017). Moreover, excessive oxidative stress is also associated with RV hypertrophy, RV dilation, and RV failure (Redout et al., 2010). The increased oxidant levels result in cardiomyocyte dysfunction and death and exacerbate RV hypertrophy (Bello-Klein et al., 2014). The final product of membrane lipid peroxidation, MDA, is a reliable biomarker for oxidative injury (Nielsen et al., 1997). In this study, the MDA content was found to be significantly increased in the RV tissues of HPH rats. Conversely, after getting treated with Tsantan Sumtang, the rats showed a significant decrease in the lipid peroxidation as compared with the hypoxia group. The free radical inhibitory potential of Tsantan Sumtang might be responsible for lowering the lipid peroxidation. SOD plays a critical role by catalyzing the dismutation of superoxide into oxygen and hydrogen peroxide. The decrease in SOD levels often leads to oxidative stress and results in vascular remodeling and pulmonary hypertension (Lakshmi Sundaram and Vasanthi, 2019). We observed that the hypoxia-induced decrease in the SOD activity was enhanced by Tsantan Sumtang treatment. Therefore, the protective effect of Tsantan Sumtang on the RV function might be related to the alleviation of oxidant damage through an increase of SOD activity. Besides, the protective effect of Tsantan Sumtang was also manifested through the increased levels of GSH and GSH-Px. GSH, a non-enzymatic antioxidant, inhibits the production of excessive oxygen free radicals (Redout et al., 2010). GSH-Px, one of the major enzymes in the glutathione redox cycle, works together with GSH to protect the body from reactive oxygen species-mediated damage and maintain the normal physiological functions of the body (Li et al., 2019). We found lower GSH and GSH-Px levels in the hypoxia group. However, after Tsantan Sumtang treatment, the GSH and GSH-Px levels significantly increased in comparison with the hypoxia group, indicating that Tsantan Sumtang promotes the redox reaction of GSH by increasing the levels of GSH and GSH-Px to protect the body from oxidant damage. GSH –Px is one of the important antioxidases, the activity of which was thought to be the degree of oxidative damage (Wu et al., 2020). The increasing level of GSH-Px means strong antioxidant effect of Tsantan Sumtang. Meanwhile, we supposed that Tsantan Sumtang may be the activator of GSH-Px, because of which the activity of GSH-Px in the 1.5 g•kg^−1^•d^−1^ group was increased to almost 2-fold of the control group (Pang et al., 2014).

We found that Tsantan Sumtang inhibits the mRNA and protein levels of ROCK in the RV of HPH rats. ROCK (Human Rho Associated Coiled Coil Containing Protein Kinase), a downstream signaling molecule of RhoGTP, is a type of serine/threonine kinase. Two homologous isomers of ROCK exist: ROCK1 and ROCK2 (Wang et al., 2019). The research showed that the activity of ROCK increases in patients with PAH, heart failure, hypertension, and stable angina pectoris (Liu et al., 2018). ROCK is also implicated in the progression of various diseases such as HPH. In the present study, Tsantan Sumtang was found to inhibit ROCK1 and ROCK2. The ROCK signal pathway is activated by caspase-3 mediated cleavage (Gabet et al., 2011). Caspase-3 is a critical marker of apoptosis; the activation of caspase-3 initiates the apoptosis of myocardial cells (Shi et al., 2019). Bax activates caspase-3 to induce apoptosis, while Bcl-2 inhibits the activity of Bax and suppresses apoptosis (Pei et al., 2019). Bcl-2 and Bax are reported to be downregulated and upregulated, respectively, under hypoxia (Shi et al., 2019). Consistent with these results, we observed a significant increase in the Bax/Bcl-2 ratio and the levels of cleaved caspase-3 and myocyte early apoptosis in the hypoxia group. Furthermore, Tsantan Sumtang treatment led to a significant increase in the expression levels of Bcl-2 and a significant decrease in the Bax/Bcl-2 ratio, caspase-3, and Bax (*p* < 0.05 vs. hypoxia group, Figures 6A, 7A,B). Using TUNEL assay, we found that the apoptosis rate significantly decreases after Tsantan Sumtang treatment (*p* < 0.05 vs. hypoxia group, Figures 4C,D). The RhoA/ROCK signaling pathway is reported to be involved in glucose-induced cardiomyocyte apoptosis (Zhou et al., 2018). The inhibitory effect of Tsantan Sumtang on the ROCK signaling pathway may be due to the strong antioxidant and anti-apoptosis properties of Tsantan Sumtang. Furthermore, the ROCK signaling pathway, in turn, regulates cardiac remodeling-related downstream transcription factors, including STAT3 and NFATc3 (Ho et al., 2012). In our study, the mRNA and protein expression levels of NFATc3 and STAT3 were found to increase under hypoxia and decrease after Tsantan Sumtang treatment (*p* < 0.05 vs. hypoxia group, Figures 6B, 7A,C).

In our previous study, we identified 35 Tsantan Sumtang constituents using UHPLC-Q-extractive hybrid quadrupole-orbitrap mass analysis (Dang et al., 2020). The constituents included nine flavonoids and their glycosides, six phenylpropanoids, ten phenolics, five terpenoids, and five other constituents. In this study, we used Tsantan Sumtang powder in which the essential oil is included. Essential oil, especially in *M. fragrans* and *S. album*, is the main bioactive ingredient. 5–15% of the essential oil is present in *M. fragrans.* This oil has strong antioxidant properties and inhibits the proliferation of hypoxia-induced pulmonary artery smooth muscle cells (PASMCs) (Ma et al., 2018). Zhang et al. found that the essential oil of *M. fragrans* alleviates the myocardium injury induced by ischemia-reperfusion and protects the cardiac function (Zhang et al., 2013; Zhang et al., 2019). The essential oil of *S. album* L. has significant antioxidant, anti-inflammatory, and anticancer properties (Huang et al., 2015). The essential oil from *S. album* was found to significantly relieve the symptoms of sudden angina pectoris to the same degree as nitroglycerin (Zhang et al., 2013). α-Santalol and β-Santalol, two main components of the essential oil of *S. album* L., possess neuro-pharmacological activities (He et al., 2019). The flavonoids of *C. axillaris* were found to protect against myocardial ischemia and myocardial infarction in rats with myocardial ischemia-reperfusion injury (Li and Cui, 2014; Li et al., 2014). Besides, these flavonoids also alleviate cardiac dysfunction and myocardial interstitial fibrosis (Li and Cui, 2014; Li et al., 2014; Sun et al., 2014). The flavonoids of *C. axillaris* possess potent antioxidant properties (Bao et al., 2001). Moreover, the organic acids from *C. axillaris* decrease the area of myocardial infarction caused by myocardial ischemia-reperfusion injury in rats. These acids also reduce lactate dehydrogenase (LDH) levels in cardiomyocytes of rats with a hypoxia-reoxygenation injury. Citric acid and l-malic acid are the main active organic acids (Tang et al., 2013; Zhou et al., 2017). Gallic acid possesses strong *in vitro* and *in vivo* antioxidant activity (Chen et al., 2015). It also prevents reperfusion injury associated with myocardial ischemia and alleviates myocardial hypertrophy and myocardial fibrosis. Due to these properties, gallic acid is considered as a potential candidate for the treatment of myocardial fibrosis and hypertrophy (Shi and Hao, 2020). Besides, isoquercitrin and quercetin were found to decrease mPAP through inhibition of proliferation of PASMCs and blockage of the plate-derived growth factor receptor β signaling pathway (Cao et al., 2017; Cheng et al., 2017). Ursolic acid inhibited lipid peroxidation and increased the activities of enzymatic antioxidants and the levels of non-enzymatic antioxidants in heart tissues (Chen et al., 2015; Wang et al., 2018). Therefore, in the present study, Tsantan Sumtang capsules (Z20020094) were used, and the powder was intragastrically administered to the rats to investigate the effect of Tsantan Sumtang on RV function and study its underlying mechanism.

## Conclusion

This study demonstrates that Tsantan Sumtang could restore RV function, improve RV-PA coupling, recover hemodynamic and hematological indexes, and protect RV against the structural maladaptive remodeling in HPH rats. The protective mechanism of Tsantan Sumtang on RV function is probably mediated by suppressing the ROCK signaling pathway and its downstream signaling molecules. The suppression of the ROCK signaling pathway may be the result of antioxidant and anti-apoptosis effects of Tsantan Sumtang.

## Data Availability Statement

The datasets presented in this study can be found in online repositories. The names of the repository/repositories and accession number(s) can be found in the article/Supplementary Material.

## Ethics Statement

The animal study was reviewed and approved by the Institutional Animal Care and Use Committee of Qinghai University.

## Author Contributions

ZY, XN, and KL participated in animal experiments, collected the experimental data, and wrote the first draft. Sun Hai Xia guided the measurements of cardiac function related indexes. GJ guided the operation of the hypobaric chamber. XJ helped with echocardiography and the measurement of cardiac function related indexes. ZL and DL designed the study and provided necessary facilities for experiments and revised the manuscript. SS contributed to the GC-MS and HPLC analysis. All authors have read and approved the final version of the manuscript.

## Funding

This work was supported by the National Natural Science Foundation of China (81660308 and 81860768), Applied Basic Research Project of Qinghai province of China (2018-ZJ-761), and the Young and Middle-Aged Foundation Team Project of Medical College of Qinghai University (2018-kyt-3). West Light Foundation of The Chinese Academy of Sciences and Natural Science Foundation of China (82060786).

## Conflict of Interest

The authors declare that the research was conducted in the absence of any commercial or financial relationships that could be construed as a potential conflict of interest.
